# miR-203 enhances let-7 biogenesis by targeting LIN28B to suppress tumor growth in lung cancer

**DOI:** 10.1038/srep42680

**Published:** 2017-02-20

**Authors:** Yong Zhou, Hongwei Liang, Zhicong Liao, Yanbo Wang, Xiuting Hu, Xi Chen, Lin Xu, Zhibin Hu

**Affiliations:** 1 Department of Epidemiology, School of Public Health, Nanjing Medical University, Nanjing, Jiangsu 210000, China; 2 The Fourth Clinical College of Nanjing Medical University, Nanjing, Jiangsu 210000, China; 3 Department of Thoracic and Cardiovascular Surgery, The Affiliated Drum Tower Hospital of Medical School of Nanjing University, Nanjing, Jiangsu 210000, China; 4 State Key Laboratory of Pharmaceutical Biotechnology, Nanjing Advanced Institute of Life Sciences, Jiangsu Engineering Research Center for MicroRNA Biology and Biotechnology, Nanjing, Jiangsu 210093, China; 5 Nanjing multi-center biobank, Nanjing, Jiangsu 210008, China

## Abstract

Human cancers often exhibit increased microRNA (miRNA) biogenesis and global aberrant expression of miRNAs; thus, targeting the miRNA biogenesis pathway represents a novel strategy for cancer therapy. Here, we report that miR-203 enhances the biogenesis of tumor suppressor let-7 in lung cancer by directly targeting LIN28B. Specially, we found that the LIN28B protein levels were dramatically increased in lung cancer tissues, but its mRNA levels did not differ significantly, suggesting that a post-transcriptional mechanism is involved in LIN28B regulation. Interestingly, miR-203 overexpression was accompanied by massive upregulation of a group of miRNAs, especially let-7, and the let-7 expression level was concordant with the miR-203 expression in lung cancer tissues, implying its biological relevance. Furthermore, we showed that miR-203 played a critical role in inhibiting the proliferation and promoting the apoptosis of lung cancer cells by suppressing LIN28B and enhancing let-7 biogenesis. In summary, our results establish a novel mechanism by which miR-203, LIN28B and let-7 are tightly linked to form a regulatory network in lung cancer cells. The findings shed light on the role of a specific miRNA as a modulator of miRNA biogenesis and provide basis for developing new strategies for lung cancer therapy.

microRNAs (miRNAs) are often dysregulated in human malignancies and can function as either oncogenes or tumor-suppressor genes^1^; therefore, they are considered to be emerging ‘hallmarks’ of cancer^2^. Notably, human cancers often exhibit abnormal miRNA biogenesis, inducing wide-ranging over or under expression of miRNAs^3^. In fact, critical components of miRNA biogenesis, including Dicer1, Drosha, TARBP2 and XPO5, have been identified as haplo-insufficient tumor suppressors^4^. Therefore, there is considerable interest in understanding the mechanisms of miRNA biogenesis, and it is thought that targeting the miRNA biogenesis pathway may lead to the development of novel cancer therapies^4^.

miRNA biogenesis consists of a series of biochemical steps, and disruption of any step can influence the miRNA abundance and contribute to tumorigenesis^4^. One of the best-studied repressors of miRNA biogenesis is Lin28, an RNA-binding protein initially identified in *Caenorhabditis elegans*, which has crucial and diverse roles in development and diseases^5^,^6^,^7^,^8^,^9^. These roles are enacted at least partially through blocking the maturation of let-7^10^,^11^,^12^. The let-7 family is one of the most abundant miRNA families in mammals, and members of this family function as tumor suppressors by targeting multiple oncogenes, including RAS, HMGA2 and c-Myc^13^,^14^.Mammals have two Lin28 homologs, LIN28A (also known as Lin28) and LIN28B, both of which play similar suppressive roles in let-7 biogenesis^15^,^16^,^17^. High levels of LIN28A/LIN28B proteins are associated with the biological behaviors and poor prognosis of many cancers^18^. Given the importance of the Lin28/let-7 regulatory circuit in development, pluripotency and tumorigenesis, it may provide novel opportunities for cancer treatment^12^. However, the mechanisms controlling the Lin28/let-7 circuit remain largely unknown.

In this study, we discovered that miR-203 directly targets LIN28B in lung cancer cells, thereby disrupting the Lin28/let-7 circuit, enhancing let-7 biogenesis and consequently inhibiting the proliferation and promoting the apoptosis of lung cancer cells. These findings not only provide novel insights into the mechanism by which miRNAs mediate tumor suppression but also offer new targets for cancer treatment.

## Results

### The upregulation of LIN28B protein, but not mRNA, in lung cancer tissues

**Figure 1:**
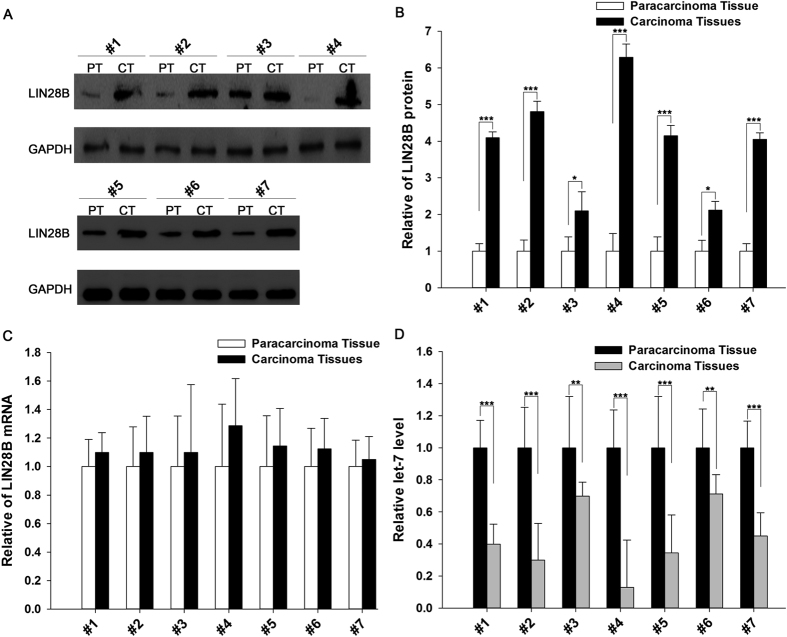
The LIN28B protein and mRNA and let-7 expression levels in NSCLC tissues. **(A** and **B)** The results of a Western blot analysis of the expression levels of the LIN28B protein in 7 pairs of cancer (CT) and normal adjacent (PT) tissue samples. (**A**) A representative image; (**B**) The results of a quantitative analysis. Full-length blots/gels are presented in Supplementary Fig. 6. **(C)** The results of a quantitative RT-PCR analysis of the relative expression levels of LIN28B mRNA in 7 pairs of CT and PT samples. **(D)** The results of a quantitative RT-PCR analysis of the relative expression levels of let-7 in 7 pairs of CT and PT samples. The results are presented as the means ± SE of three independent experiments (^*^P < 0.05, ^**^P < 0.01, ^***^P < 0.001).

We first determined the expression patterns of LIN28B in non-small cell lung cancer (NSCLC) tissues. After measuring the expression levels of the LIN28B protein in seven pairs of NSCLC tissues and corresponding normal adjacent tissues via Western blotting (the clinical features of these tissue samples are listed in Table S1), we observed that the LIN28B protein levels were significantly higher in the cancer tissues (Fig. 1A and B). Subsequently, we performed quantitative RT-PCR to measure the expression levels of LIN28B mRNA in the same 7 pairs of cancerous and noncancerous tissues. It was observed that the LIN28B mRNA levels did not differ significantly between the cancerous and noncancerous tissues (Fig. 1C). The disparity between the LIN28B protein and mRNA levels in NSCLC strongly suggests that a post-transcriptional mechanism is involved in the regulation of LIN28B. Additionally, we found that the expression levels of let-7 were conspicuously decreased in the cancer tissues compared with those in normal adjacent tissues (Fig. 1D), which is consistent with the previous findings that Lin28B blocks the maturation of let-7 miRNAs^10^,^11^,^12^.

### Identification of conserved miR-203 target sites within the 3′-UTR of LIN28B

One important mode of post-transcriptional regulation is the repression of mRNA transcripts by miRNAs. miRNAs are therefore likely to play a biologically relevant role in regulating LIN28B expression in NSCLC. Three computational algorithms, including TargetScan^19^, miRanda^20^ and RNAhydrid^21^, were used in combination to identify potential miRNAs that can target LIN28B. A total of 13 miRNAs, including miR-203, miR-30, let-7, miR-132, miR-181, miR-212, miR-101, miR-9, miR-125, miR-98, miR-196, miR-23 and miR-499, were identified as candidate miRNAs by all three computational algorithms (Table S2). Because miRNAs are generally thought to have expression patterns that are opposite to those of their targets^22^,^23^, we next investigated whether these miRNAs were inversely correlated with the LIN28B level in NSCLC. After determining the expression levels of these miRNAs in the same 7 pairs of NSCLC tissues and normal adjacent tissues, we observed that 8 miRNAs (miR-203, miR-30, let-7, miR-132, miR-181, miR-212, miR-101 and miR-9) were downregulated in the NSCLC tissues, while the other 5 miRNAs (miR-125, miR-98, miR-196, miR-23 and miR-499) were upregulated (Fig. S1). The notion that LIN28B is the direct target of let-7 has already been established^24^,^25^. In addition to let-7, miR-181^26^, miR-9^27^,^28^, miR-30^29^, miR-101^30^, miR-212^31^ and miR-132^32^,^33^ have also been shown to be correlated with LIN28B and may repress the translation of this protein (Table S2).

**Figure 2:**
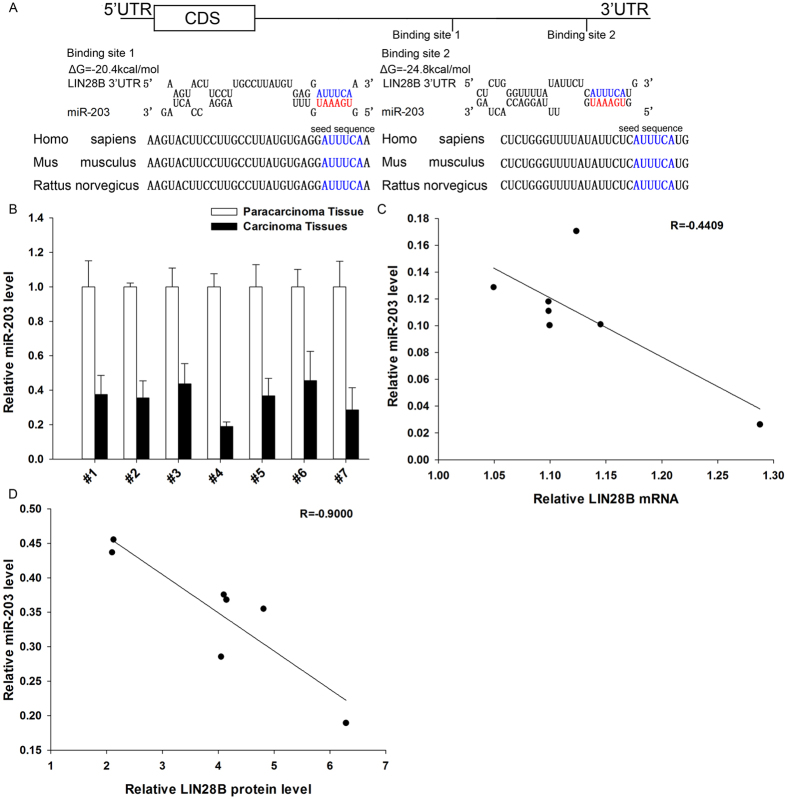
Detection of an inverse correlation between the miR-203 and LIN28B levels in NSCLC tissue samples. **(A)** A schematic depicting the hypothetical duplexes formed through an interaction between the binding sites in the LIN28B 3′-UTR (top) and miR-203 (bottom). The predicted free energy of each hybrid is indicated. The seed recognition sites are denoted, and all nucleotides in these regions are highly conserved across species. **(B)** The results of a quantitative RT-PCR analysis of the miR-203 expression levels in 7 pairs of CT and PT samples. **(C)** A Pearson’s correlation scatter plot of the fold-change in the levels of miR-203 and LIN28B protein in human NSCLC tissues. **(D)** A Pearson’s correlation scatter plot of the fold-change in the levels of miR-203 and LIN28B mRNA in human NSCLC tissues. The results are presented as the means ± SE of three independent experiments (^*^P < 0.05, ^**^P < 0.01, ^***^P < 0.001).

We next investigated whether miR-203 can directly target LIN28B in NSCLC. As shown in Fig. 2A, we observed two potential miR-203 binding sites within the 3′-UTR of LIN28B mRNA. The minimum free energy values of the hybrids between miR-203 and LIN28B mRNA were −20.4 and −24.8 kcal/mol, which are well within the range of previously confirmed miRNA-target pairs. Moreover, there was perfect base pairing between the seed regions (the core sequence that encompasses the first 2–8 bases of the mature miRNA) and the cognate targets, and the miR-203 binding sequences in the LIN28B 3′-UTR were highly conserved across species (Fig. 2A).

Subsequently, we determined the expression levels of miR-203 in the same 7 pairs of NSCLC tissues and corresponding noncancerous tissues. As expected, miR-203 was observed to be significantly downregulated in NSCLC tissues (Fig. 2B). In addition, the expression of miR-203 correlated more negatively with the LIN28B protein levels compared with the LIN28B mRNA levels, as illustrated with Pearson’s correlation scatter plots (Fig. 2C and D). These results are consistent with the theory that animal miRNAs block translation without affecting the transcript levels^23^. Thus, based on computational predictions and the inverse correlation between miR-203 and LIN28B protein levels in NSCLC, we inferred that LIN28B expression was regulated by a miR-203-mediated post-transcriptional mechanism.

### miR-203 enhances let-7 biogenesis by directly targeting LIN28B

**Figure 3:**
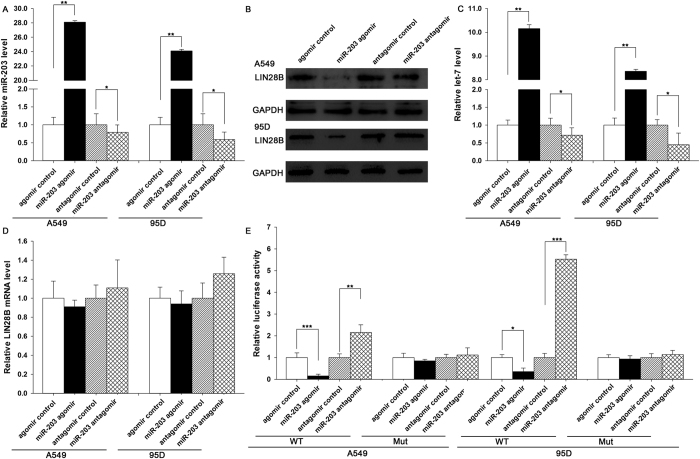
Direct post-transcriptional regulation of LIN28B expression through miR-203. **(A)** The results of a quantitative RT-PCR analysis of the miR-203 levels in A549 and 95D cells treated with the control agomir, miR-203 agomir, control antagomir or miR-203 antagomir. **(B** and **C)** The results of a Western blot analysis of the LIN28B protein levels in A549 and 95D cells treated with the control agomir, miR-203 agomir, control antagomir or miR-203 antagomir. (**B**) A representative image; Full-length blots/gels are presented in Supplementary Fig. 6. (**C**) The results of a quantitative analysis. **(D)** The results of a quantitative RT-PCR analysis of the LIN28B mRNA levels in A549 and 95D cells treated with the control agomir, miR-203 agomir, control antagomir or miR-203 antagomir. **(E)** Firefly luciferase reporters containing either wild-type (WT) or mutant (Mut) miR-203 binding sites in the LIN28B 3′-UTR were co-transfected into A549 and 95D cells along with control agomir, miR-203 agomir, control antagomir or miR-203 antagomir. The cells were assayed using a luciferase assay kit 24 h post-transfection. The results are presented as the means ± SE of three independent experiments (^*^P < 0.05, ^**^P < 0.01, ^***^P < 0.001).

The correlation between miR-203 and LIN28B was further examined by evaluating LIN28B expression in the A549 and 95D human NSCLC cell lines after overexpression or knockdown of miR-203. In these experiments, the overexpression of miR-203 was achieved by transfecting cells with a miR-203 agomir, which was a synthetic RNA oligonucleotide that mimics the miR-203 precursor. Knockdown of miR-203 was achieved by transfecting cells with a miR-203 antagomir, which was a chemically modified antisense oligonucleotide designed to specifically target mature miR-203. The efficient overexpression and knockdown of miR-203 in A549 and 95D cells are shown in Fig. 3A. The cellular miR-203 levels increased by approximately 20-fold when A549 and 95D cells were transfected with the miR-203 agomir, and these levels dropped to approximately 50% of the normal levels when the A549 and 95D cells were treated with the miR-203 antagomir. As anticipated, the overexpression of miR-203 significantly suppressed the LIN28B protein levels in A549 and 95D cells (Fig. 3B). Consequently, the expression levels of let-7 were decreased (Fig. 3C), suggesting that the induction of miR-203 inhibits LIN28B expression and subsequently rescues the suppression of let-7 by LIN28B. On the other hand, the expression of the LIN28B protein was increased in A549 and 95D cells transfected with the miR-203 antagomir (Fig. 3B), leading to the subsequent downregulation of let-7 in A549 and 95D cells (Fig. 3C). To determine the regulatory level at which miR-203 influenced LIN28B expression, we repeated the experiments above and examined the expression of LIN28B mRNA after transfection. Neither overexpression nor knockdown of miR-203 affected the LIN28B mRNA levels in either cell line (Fig. 3D). These results demonstrated that miR-203 specifically represses LIN28B protein at the post-transcriptional level to enhance let-7 biogenesis.

To determine whether the negative regulatory effects of miR-203 on LIN28B expression were mediated through the binding of miR-203 to the presumed sites in the 3′-UTR of the LIN28B mRNA, the full length 3′-UTR of LIN28B containing the two presumed miR-203 binding sites was placed downstream of the firefly luciferase gene in a reporter plasmid. The resulting plasmid was transfected into A549 and 95D cells along with a transfection control plasmid (β-gal) and one of the following: miR-203 agomir, miR-203 antagomir, or scrambled negative control RNA. As expected, the luciferase activity was intensively reduced in cells transfected with the miR-203 agomir (Fig. 3E). In contrast, the luciferase activity was significantly increased in cells transfected with the miR-203 antagomir (Fig. 3E). Furthermore, we introduced point mutations into the corresponding complementary sites in the 3′-UTR of LIN28B to eliminate the predicted miR-203 binding sites. Neither overexpression nor knockdown of miR-203 changed the luciferase activity of the mutant luciferase reporter (Fig. 3E). These findings suggested that the miRNA binding sites strongly contributed to the miRNA-mRNA interaction. Together, our results indicate that miR-203 directly recognizes and binds to the 3′-UTR of the LIN28B mRNA transcript and suppresses LIN28B expression, which in turn enhances let-7 biogenesis in lung cancer cells.

### miR-203 inhibits the proliferation and promotes the apoptosis of lung cancer cells by suppressing LIN28B and enhancing let-7 biogenesis

**Figure 4:**
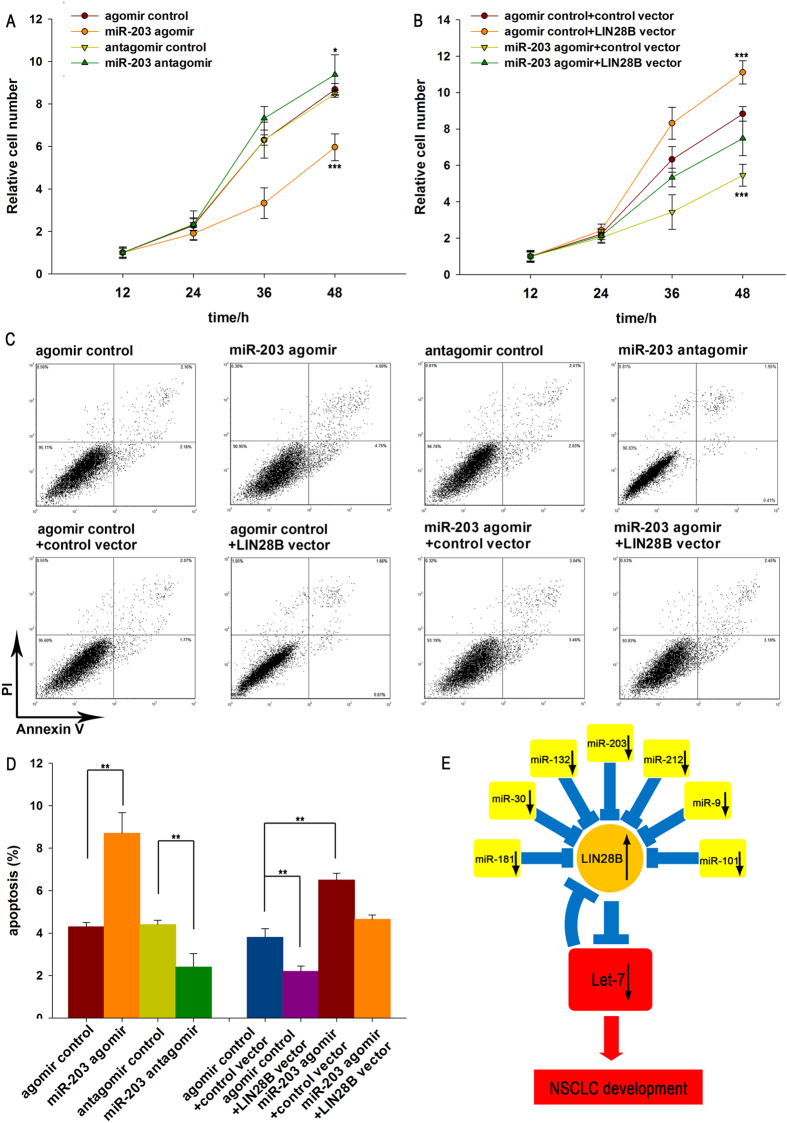
The effects of LIN28B targeting via miR-203 on the proliferation and apoptosis of A549 cells. **(A)** The relative proliferation of A549 cells after transfection with equal doses of control agomir, miR-203 agomir, control antagomir or miR-203 antagomir. **(B)** The relative proliferation of A549 cells after transfection with equal doses of control agomir plus control vector, miR-203 agomir plus control vector, control agomir plus LIN28B vector, or miR-203 agomir plus LIN28B vector. **(C** and **D)** A549 cells were transfected with equal doses of control agomir, miR-203 agomir, control antagomir or miR-203 antagomir, or with equal doses of control agomir plus control vector, miR-203 agomir plus control vector, control agomir plus LIN28B vector, or miR-203 agomir plus LIN28B vector. The cell apoptosis profiles were analyzed using flow cytometry. The biparametric histogram shows the cells in early (bottom right quadrant) and late apoptotic states (upper right quadrant). Viable cells are double negative (bottom left quadrant). (**C**) A representative image; (**D**) The results of a quantitative analysis. **(E)** A model of the miRNAs-mediated enhancement of let-7 biogenesis by targeting LIN28B to suppress tumor growth in lung cancer. The results are presented as the means ± SE of three independent experiments (^*^P < 0.05, ^**^P < 0.01, ^***^P < 0.001).

Next, we analyzed the biological consequences of LIN28B suppression mediated by miR-203 in lung cancer cells. In support of the notion that miR-203 functions as a key tumor-suppressive miRNA in NSCLC^34^,^35^,^36^, A549 cells transfected with the miR-203 agomir exhibited diminished proliferation; in contrast, knocking down miR-203 had the opposite effect on cell proliferation (Fig. 4A). We also investigated the effects of miR-203 on lung cancer cell apoptosis via a flow cytometric analysis. The results showed that the percentage of apoptotic cells was significantly higher among the A549 cells transfected with the miR-203 agomir but was lower among the A549 cells transfected with the miR-203 antagomir (Fig. 4C and D).

We also assessed the role of LIN28B in cell proliferation and apoptosis after the overexpression or silencing of LIN28B in lung cancer cells. To knock down LIN28B expression, a siRNA sequence targeting human LIN28B cDNA was designed and transfected into A549 cells. To overexpress LIN28B, a plasmid expressing the LIN28B ORF without the miR-203-responsive 3′-UTR was transfected into A549 cells. The efficient overexpression and silencing of LIN28B was verified, and the data are shown in Fig. S2. Our findings are consistent with previous studies showing that LIN28B inhibits let-7 biogenesis, which in turn promotes the proliferation and inhibits the apoptosis of cancer cells^24^,^25^, A549 cells transfected with LIN28B siRNA had a significantly lower proliferation rate and a higher apoptosis rate, whereas the cells transfected with the LIN28B overexpression plasmid showed the opposite effects (Fig. S2). Thus, miR-203 and its target, LIN28B, had opposite expression patterns and biological functions in lung cancer cells.

To investigate whether the regulation of cell proliferation and apoptosis by miR-203 is executed in a LIN28B-dependent manner, we co-transfected A549 cells with the miR-203 agomir and a LIN28B overexpression plasmid. Compared to the cells transfected with the miR-203 agomir alone, the cells co-transfected with the miR-203 agomir and the LIN28B overexpression plasmid exhibited a significantly increased proliferation rate (Fig. 4B), suggesting that miR-203-resistant LIN28B rescued the suppression of LIN28B expression induced by miR-203 and attenuated the anti-proliferative effect of miR-203. Furthermore, when A549 cells were simultaneously transfected with the miR-203 agomir and the LIN28B overexpression plasmid, the pro-apoptotic effect of miR-203 was dramatically attenuated (Fig. 4C and D). These results indicated that miR-203 might modulate cell proliferation and apoptosis by downregulating LIN28B in lung cancer cells.

Finally, we investigated whether miR-203, LIN28B and let-7 are tightly linked to form a regulatory network in lung cancer cells. A549 cells in which LIN28B expression was silenced using siRNA had a much higher level of let-7, whereas the cells transfected with the LIN28B overexpression plasmid showed decreased let-7 (Fig. S2). These results suggest that LIN28B functions as a link between the miRNAs miR-203 and let-7. We next investigated whether the overexpression or knockdown of miR-203 influenced cell proliferation and apoptosis by affecting let-7 biogenesis. According to previous reports, let-7 is a key tumor-suppressive miRNA in NSCLC^37^,^38^,^39^,^40^. As expected, A549 cells transfected with the let-7 agomir exhibited decreased proliferation and increased apoptosis; in contrast, knockdown of let-7 had the opposite effects on A549 cells (Fig. S4). Compared to the cells transfected with the miR-203 agomir, the cells co-transfected with both the miR-203 agomir and let-7 antagomir exhibited a significantly increased proliferation rate and decreased apoptosis (Fig. S4), suggesting that miR-203 inhibits the proliferation and promotes the apoptosis of lung cancer cells by suppressing LIN28B and enhancing let-7 biogenesis. In summary, the present findings indicate that LIN28B is crucial for the proliferation and invasion of lung cancer cells due to its suppression of let-7 biogenesis and that miR-203 enhances let-7 biogenesis by silencing LIN28B expression, and consequently functions as a critical tumor suppressor during lung tumorigenesis.

## Discussion

Recent studies have discovered that LIN28 and let-7 family miRNAs tend to play opposing roles in many cellular processes, in particular those involved in cancer development and progression^12^. Indeed, LIN28B and let-7 are inversely expressed in normal and malignant tissues^9^,^41^. LIN28B negatively regulates let-7 family miRNAs via its RNA-binding domains (RBDs), which include a cold-shock domain (CSD) at the N-terminus and two Cys-Cys-His-Cys (CCHC)-type zinc finger domains at the C-terminus^42^,^43^,^44^,^45^. Both the CSD and CCHC zinc fingers of LIN28B can interact with the conserved residues of pri-let-7 and pre-let-7; the CSD inserts into the apical point of the precursor loop, while the CCHC zinc fingers dimerize on a GGAG motif adjacent to the Dicer cleavage site^46^,^47^. The binding of LIN28B to either pri-let-7 or pre-let-7 inhibits let-7 precursor processing by Drosha and Dicer^48^. Upon binding to pre-let-7, LIN28B recruits TUT4/TUT7, which causes oligo-uridylation at the 3′ terminal of pre-let-7^15^,^49^,^50^. Under normal conditions, Dicer recognizes the two nucleotides at the 3′ terminal via its PAZ domain; however, oligo-uridylation elongates the 3′ terminal, resulting in resistance to Dicer cleavage. Oligo-uridylated pre-let-7 can also be degenerated by the 3′-5′ exonuclease Dis312^51^,^52^. Thus, LIN28B not only inhibits the biogenesis of let-7 family miRNAs but also induces their degradation. Furthermore, high levels of LIN28B proteins are associated with the biological behaviors and poor prognosis of many cancers. The presence of a double-negative feedback loop between LIN28A/LIN28B and let-7 was also reported^12^. In addition to let-7, miR-181^26^, miR-30^29^, miR-9^27^,^28^, miR-132^32^,^33^, miR-101^30^ and miR-212^31^ have also been shown to directly bind the 3′-UTR of LIN28B and repress the translation of this protein. Because these miRNAs are frequently under-expressed in malignant tumors, higher levels of LIN28 expression are frequently observed in tumors, including lung cancer (Table S2). Here we showed that the overexpression of miR-203 results in increased expression of let-7, and knockdown of let-7 reversed the inhibitory effects of miR-203 overexpression on tumor cell growth. Mechanistically, miR-203 directly targets LIN28B, which is a critical repressor of the maturation of miRNAs, particularly let-7. Previous studies have defined a regulatory loop consisting of Lin28 and let-7, in which LIN28B suppresses let-7 maturation and let-7, in turn, directly targets LIN28B^21^,^22^. In this study, let-7 expression was found to be concordant with the miR-203 expression in normal and tumor tissues, underscoring the coordinated regulation of these two miRNAs via LIN28B as a link. In fact, let-7 has been regarded as a bona fide tumor suppressor, and accumulating evidence has demonstrated that it has crucial roles in the development of cancer. For example, let-7 targets multiple oncogenes, including RAS, HMGA2 and c-Myc^13^,^14^,^53^,^54^,^55^. Recently, let-7 has been shown to act in a metastasis-associated signaling cascade involving the RAF kinase inhibitory protein^56^,^57^. Accordingly, miR-203-induced let-7 provides a conserved mechanism to explain the suppressive role of miR-203 during lung tumorigenesis.

It has long been known that miR-203 acts as a tumor-suppressive miRNA in many cancer types^35^,^36^,^58^,^59^,^60^,^61^,^62^. It was also shown to be downregulated in various prostate cancer cell lines^58^. The ectopic expression of miR-203 in prostate cancer cell lines could influence their proliferation, apoptosis, and migration^58^,^59^, and the overexpression of miR-203 in laryngeal carcinoma cells reduced the cell viability and led to cell cycle arrest in the G1 phase^61^. Additionally, the expression of miR-203 suppressed cell proliferation and migration in human triple-negative breast cancer cells^62^. Because a single miRNA can target multiple genes, and multiple miRNAs can target a single gene, we cannot rule out the possibility that additional targets are simultaneously affected by miR-203. Therefore, at this stage, it is important to investigate how critical the newly identified pathway composed of miR-203, LIN28B and let-7 is in lung tumorigenesis. In this study, we found that miR-96 can inhibit the proliferation and promote the apoptosis of lung cancer cells and that LIN28B silencing can mimic the miR-203-induced cellular phenotypes. More importantly, restoration of LIN28B expression with a miR-203-resistant LIN28B overexpression plasmid completely reversed the miR-203-induced cellular phenotypes, suggesting that targeting LIN28B is a major mechanism by which miR-203 exerts its tumor-suppressive functions. Therefore, modulation of LIN28B by miR-203 may explain, at least in part, why the downregulation of miR-203 during lung tumorigenesis can promote tumor growth.

Taken together, the findings of this study show that miR-203 directly targets LIN28B and enhances let-7 biogenesis to suppress tumor growth in lung cancer. These findings provide new insights into a potential application for miRNA-mediated tumor suppression through enhancing miRNA biogenesis, which may serve as novel therapeutic approach for cancer.

## Materials and Methods

### Ethics Statement

All methods and experimental protocols were approved by Nanjing Medical University and were carried out in accordance with the approved guidelines. Written consent was provided by all of the patients, and the Ethics Committee of the Affiliated Gulou Hospital of Nanjing University approved all aspects of this study. Informed consent was obtained from all subjects enrolled in the studies that provided the samples. NSCLC tissues and paired normal adjacent tissues were derived from patients undergoing a surgical procedure at the Affiliated Gulou Hospital of Nanjing University (Nanjing, China). Both tumors and noncancerous tissues were confirmed histologically. The pathological type of each cancer was determined to be infiltrating ductal carcinoma. Tissue fragments were immediately frozen in liquid nitrogen at the time of surgery and stored at −80 °C. The clinical features of the patients are listed in Table S1.

### Cell culture

The human lung cancer cell lines, A549 and 95D, were cultured in DMEM supplemented with 10% fetal bovine serum (FBS, GIBCO, CA, USA). All cells were incubated in a 5% CO_2_ incubator at 37 °C in a water-saturated atmosphere.

### RNA isolation and quantitative RT-PCR

Total RNA was extracted from the cultured cells and tissues using the TRIzol Reagent (Invitrogen) according to the manufacturer’s instructions. Human subjects who provided the tissues and cells have obtained the informed consent. Assays to quantify mature miRNAs were performed using Taqman microRNA probes (Applied Biosystems, Foster City, CA) according to the manufacturer’s instructions. Briefly, 1 μg of total RNA was reverse-transcribed to cDNA using AMV reverse transcriptase (TaKaRa, Dalian, China) and a stem-loop RT primer (Applied Biosystems). The reaction conditions were as follows: 16 °C for 30 min, 42 °C for 30 min and 85 °C for 5 min. Real-time PCR was performed using a TaqMan PCR kit and an Applied Biosystems 7300 Sequence Detection System (Applied Biosystems). The reactions were incubated in a 96-well optical plate at 95 °C for 10 min, followed by 40 cycles of 95 °C for 15 s and 60 °C for 1 min. All of the reactions were run in triplicate. After the reactions were complete, the cycle threshold (C_T_) data were determined using fixed threshold settings, and the mean C_T_ was determined from triplicate PCR assays. A comparative C_T_ method was used to compare each condition to the control reactions. U6 snRNA was used as an internal control, and the relative amount of miRNA normalized to U6 was calculated with the equation 2−ΔΔC_T_, in which ΔΔC_T_ = (C_T_ miRNA − C_T_ U6) target − (C_T_ miRNA − C_T_ U6) control.

To quantify the LIN28B and GAPDH mRNA, 1 μg of total RNA was reverse-transcribed to cDNA using Oligo d(T)18 primers (TaKaRa) and ThermoScript reverse transcriptase (Invitrogen). The reaction conditions were as follows: 42 °C for 60 min and 70 °C for 10 min. Real-time PCR was then performed with the RT product, and these reactions included SYBR Green dye (Invitrogen) and specific primers for LIN28B and GAPDH. The sequences of the primers were as follows: LIN28B (sense): 5′-CATGGTGGCAAACTGCCCACATAA-3′; LIN28B (antisense): 5′-TTCGTGGAGGAAGCTTCTTGAGGT-3′; GAPDH (sense): 5′-GATATTGTTGCCATCAATGAC-3′; and GAPDH (antisense): 5′-TTGATTTTGGAGGGATCTCG-3′. The reactions were incubated at 95 °C for 5 min, followed by 40 cycles of 95 °C for 30 s, 60 °C for 30 s and 72 °C for 30 s. After the reactions were complete, the C_T_ values were determined by setting a fixed threshold. The relative amount of LIN28B mRNA was normalized to GAPDH.

### miRNA overexpression or knockdown

miRNA overexpression was achieved by transfecting cells with a miRNA agomir, which is a chemically modified synthetic RNA oligonucleotide duplex mimicking an miRNA precursor, with modification of the antisense chain, a 3′ end cholesterol modification, 5′ end two thio skeleton modification, 3′ end four thio skeleton modification, and the whole chain was modified by a methylene group. Knockdown was achieved by transfecting cells with a miRNA antagomir, which is a chemically modified single-stranded antisense oligonucleotide designed to specifically target mature miRNA, with a 3′ end cholesterol modification, 5′ end two thio skeleton modification, 3′ end four thio skeleton modification, and the whole chain was modified by a methylene group. Synthetic RNA molecules were purchased from GenePharma (Shanghai, China). A549 and 95D cells were seeded in 6-well plates and transfected with Lipofectamine 2000 (Invitrogen) on the following day when the cells were approximately 70% confluent. For the overexpression of miRNAs, 10 pmol of miR-203 agomir or let-7 agomir were used. For the miRNA knockdown, 10 pmol of miR-203 antagomir or let-7 antagomir were used. After 6 h, the medium was changed to DMEM that was supplemented with 10% fetal bovine serum. The cells were harvested 24 h or 48 h after the transfection for the isolation of total RNA or protein, respectively.

### Plasmid construction and siRNA interference assay

A mammalian expression plasmid (pReceiver-M02-LIN28B) designed to specially express the full-length open reading frame (ORF) of human LIN28B without the miR-203–responsive 3′-UTR was purchased from GeneCopoeia (Germantown, MD, USA). An empty plasmid served as a negative control. The siRNAs (sequence#1: 5′-CATAACAGGTCTTCTTCATAT-3′; sequence#2: 5′-GCAGAGAUCUCAGAACGGUUU-3′) targeting human LIN28B were designed and synthesized by GenePharma (Shanghai, China). A scrambled siRNA (GenePharma) was included as a negative control. The overexpression plasmid or siRNA was transfected into A549 cells using Lipofectamine 2000 (Invitrogen) according to the manufacturer’s instructions. Total RNA or protein was isolated 24 or 48 h after transfection. The LIN28B mRNA and protein expression levels were assessed by quantitative RT-PCR and Western blotting.

The siRNA sequences targeting different sites of LIN28B were designed according to previous reports (*Cheng, SW. et al*. *Plos one 2013*) and the sequence (5′-GCAGAGAUCUCAGAACGGUUU-3′) with the best interfering effect for further experiments was selected, which has been optimized to ensure that the off-target effect is minimal.

### Luciferase reporter assay

The entire 3′-UTR of human LIN28B was amplified by PCR using human genomic DNA as a template. The PCR products were inserted into the p-MIR-reporter plasmid (Ambion, Austin, TX, USA). The insertion was confirmed to be correct by DNA sequencing. To test the binding specificity, the sequences that interact with the seed sequence of miR-203 were mutated (from AUUUCA to UAAAGU), and the mutant LIN28B 3′-UTR was inserted into an equivalent luciferase reporter plasmid. For the luciferase reporter assays, A549 and 95D cells were seeded in 6-well plates and co-transfected with 2 μg of firefly luciferase reporter plasmid, 2 μg of β-galactosidase (β-gal) expression plasmid (Ambion), and 10 pmol of miR-203 agomir, miR-203 antagomir, or scrambled negative control RNA using Lipofectamine 2000 (Invitrogen). A β-gal plasmid was used as a transfection control. Cells were harvested 24 h after transfection and were analyzed for luciferase activity using a luciferase assay kit (Promega, Madison, WI, USA).

### Protein isolation and Western blotting

Cells or tissues were lysed in RIPA lysis buffer (50 mM Tris–HCl, 150 mM NaCl, 0.1% SDS, 1% NP-40, 0.25% sodium deoxycholate and 1 mM EDTA, pH 8.0) with freshly added protease inhibitor cocktail (Roche) for 30 min on ice and were then centrifuged at 16,000 × g at 4 °C for 10 min. The supernatant was collected, and the protein concentration was calculated with a BCA protein assay kit (Thermo Scientific, Rockford, IL, USA). Proteins were separated by SDS-PAGE (Bio-Rad). After electrophoresis, the proteins were electrotransferred onto PVDF membranes (Bio-Rad) and then blocked with 5% skim milk for 1 h. The membranes were then incubated with primary antibodies at 4 °C for 12 h. After three washes in TBST, the membranes were incubated with horseradish peroxidase-conjugated secondary antibody for 1 h at room temperature. After three washes, the membranes were incubated with the SuperSignal West Pico chemiluminescence substrate (Pierce). The protein levels were normalized by probing the same blots with an anti-GAPDH antibody. The antibodies were purchased from the following sources: anti-LIN28B (F-21): (sc-130802, Santa Cruz Biotechnology, CA, USA) and anti-GAPDH (sc-365062, Santa Cruz Biotechnology, CA, USA). Protein bands were analyzed using the ImageJ software.

### Cell proliferation assay

A549 cells were plated at 5 × 10^4^ cells per well in 96-well plates and then were incubated overnight in DMEM supplemented with 10% FBS. Cells were collected 12, 24, 36 and 48 hours post-transfection. After transfection, 20 μL of 3-(4,5-dimethylthiazol-2-yl)-2,5-diphenyl tetrazolium bromide (MTT) (5 mg/mL) was added into a corresponding test well, followed by incubation for 4 h. The supernatant was then discarded, and 150 μL of DMSO was added to each well to dissolve the precipitate. The absorbance was measured at 490 nm.

### Apoptosis assays

The apoptosis of A549 cells was tested using an Annexin V-FITC/PI staining kit (BD Biosciences, CA, USA). A549 cells were cultured in 12-well plates with serum-containing complete medium. After 6 h of transfection using Lipofectamine 2000 (Invitrogen), the A549 cells were cultured with serum-depleted medium to induce apoptosis. After 24 h, the cells were washed with cold PBS and resuspended in binding buffer (100 mM HEPES, pH 7.4, 100 mM NaCl, and 25 mM CaCl2), followed by staining with Annexin V-FITC/PI at room temperature in the dark for 15 min. Apoptotic cells were then evaluated by gating the PI- and Annexin V-positive cells using a fluorescence-activated cell-sorting (FACS) flow cytometer (BD Biosciences, San Jose, CA). All experiments were performed in triplicate.

### Statistical analysis

All of the Western blotting images are representative of at least three independent experiments. Quantitative RT-PCR, the luciferase reporter assay, and the cell viability and apoptosis assays were performed in triplicate, and each experiment was repeated several times. The data shown are the means ± SE of at least three independent experiments. The differences were considered statistically significant at *p* < 0.05 determined using Student’s *t*-test.

## Additional Information

**How to cite this article**: Zhou, Y. *et al*. miR-203 enhances let-7 biogenesis by targeting LIN28B to suppress tumor growth in lung cancer. *Sci. Rep.* **7**, 42680; doi: 10.1038/srep42680 (2017).

**Publisher's note:** Springer Nature remains neutral with regard to jurisdictional claims in published maps and institutional affiliations.

## Supplementary Material

Supplementary Materials

Supplementary Table S3
