# Supplementary material for: miR-203 enhances let-7 biogenesis by targeting LIN28B to suppress tumor growth in lung cancer
Source: Sci Rep. 2017 Feb 20;7:42680. doi: 10.1038/srep42680 (PMC5316988; doi:10.1038/srep42680)
Supplement: Supplementary Materials [file srep42680-s1.doc]

**miR-203 enhances Let-7 biogenesis by targeting Lin28B to suppress tumor growth in lung cancer**

Yong Zhou^1,2,3,#^, Hongwei Liang^4, #^, Zhicong Liao^3,#^, Yanbo Wang^4^, Xiuting Hu^4^, Xi Chen^4,*^, Lin Xu^2,*^ , Zhibin Hu^1,*^

^1^Department of Epidemiology, School of Public Health, Nanjing Medical University; ^2^The Fourth Clinical College of Nanjing Medical University; ^3^Department of Thoracic and Cardiovascular Surgery, The Affiliated Drum Tower Hospital of Medical School of Nanjing University , ^4^State Key Laboratory of Pharmaceutical Biotechnology, Nanjing Advanced Institute of Life Sciences, Jiangsu Engineering Research Center for MicroRNA Biology and Biotechnology, Nanjing, Jiangsu 210093, China; ^5^Nanjing multi-center biobank, Nanjing, Jiangsu 210008, China.

**Table S1. Demographic information of NSCLC patient cohort.**

|  | Age | Gender | Tumor subtype | Pathological Stage |
| --- | --- | --- | --- | --- |
| Case #1 | 50 | Male | NSCLC | IIIA |
| Case #2 | 66 | Male | NSCLC | IIB |
| Case #3 | 49 | Male | NSCLC | IIIA |
| Case #4 | 53 | Male | NSCLC | IIA |
| Case #5 | 78 | Female | NSCLC | IIIA |
| Case #6 | 64 | Female | NSCLC | IIB |
| Case #7 | 68 | Female | NSCLC | IIB |

**Table S2.** **Candidate miRNAs predicted to target LIN28B by three computational algorithms, including TargetScan, miRanda and RNAhydrid.**

| miRNA name | targetScan | picTar | miRanda | Expression in NSCLC |
| --- | --- | --- | --- | --- |
| miR-203 | + | + | + | down-regulated |
| miR-30 | + | + | + | down-regulated |
| let-7 | + | + | + | down-regulated |
| miR-132 | + | + | + | down-regulated |
| miR-181 | + | + | + | down-regulated |
| miR-212 | + | + | + | down-regulated |
| miR-101 | + | + | + | down-regulated |
| miR-9 | + | + | + | down-regulated |
| miR-125 | + | + | + | up-regulated |
| miR-98 | + | + | + | up-regulated |
| miR-196 | + | + | + | N/A |
| miR-23 | + | + | + | N/A |
| miR-499 | + | + | + | N/A |


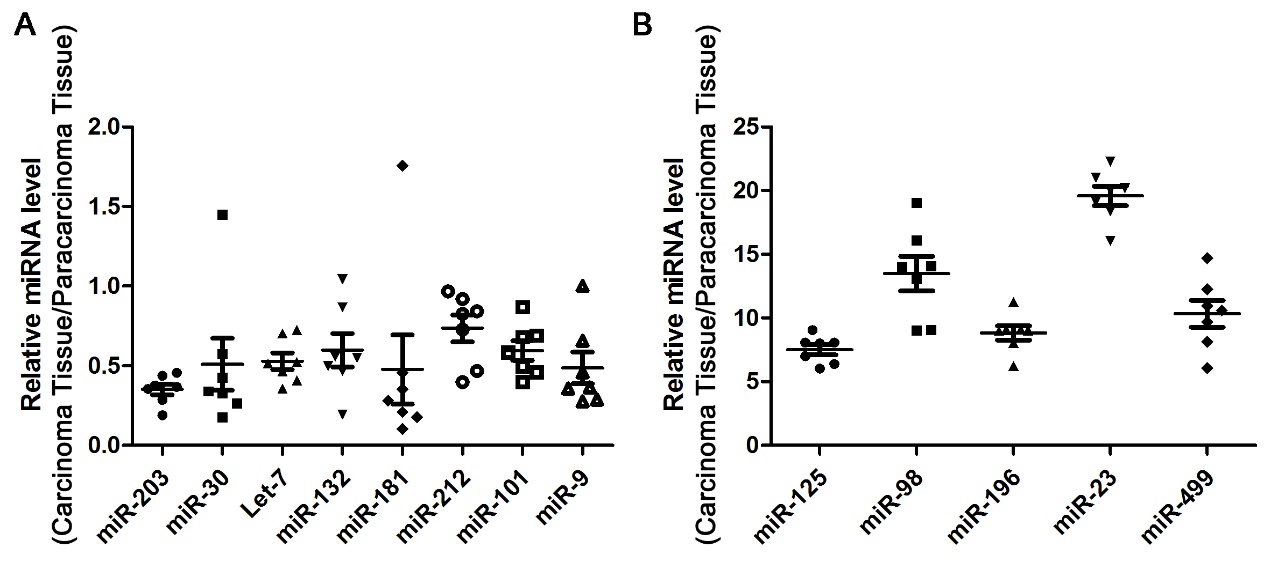


**Figure S1.** Quantitative RT-PCR analysis of expression levels of the candidate miRNAs predicted to target LIN28B in 7 pairs of NSCLC samples (A: candidate miRNAs down-regulated in cancerous tissues; B: candidate miRNAs up-regulated in cancerous tissues). The results are presented as the mean ± SE of three independent experiments (*P<0.05, **P<0.01, ***P<0.001).


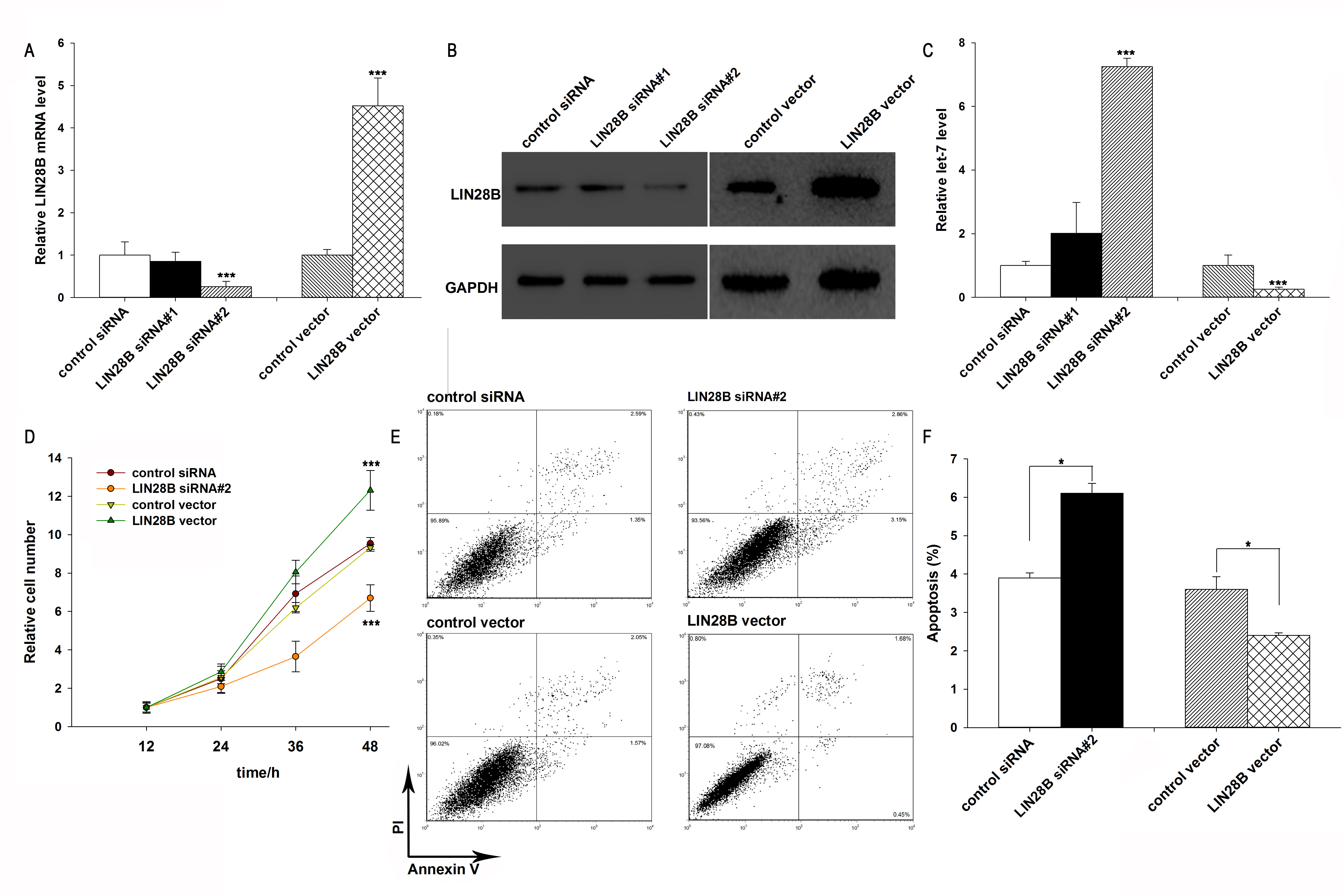


**Figure S2. Downregulation of LIN28B through siRNA and upregulation of LIN28B using an overexpression vector in A549 cells. (A)** The results of a quantitative RT-PCR analysis of the LIN28B mRNA levels in A549 cells treated with control siRNA, LIN28B siRNA, control vector or LIN28B vector. **(B and C)** The results of a Western Blot analysis of the LIN28B protein levels in A549 cells treated with control siRNA, LIN28B siRNA, control vector or LIN28B vector. B: representative image; C: The results of a quantitative analysis. **(D)** The relative proliferation of A549 cells after transfection with equal doses of control siRNA, LIN28B siRNA, control vector or LIN28B vector. **(E and F)** A549 cells were transfected with equal doses of control siRNA, LIN28B siRNA, control vector or LIN28B vector. The cell apoptosis profiles were analyzed using flow cytometry. The biparametric histogram shows cells in early (bottom right quadrant) and late apoptotic states (upper right quadrant). Viable cells are double negative (bottom left quadrant). E: The results of a quantitative analysis; F: representative image. The results are presented as the mean ± SE of three independent experiments (*P<0.05, **P<0.01, ***P<0.001).

**
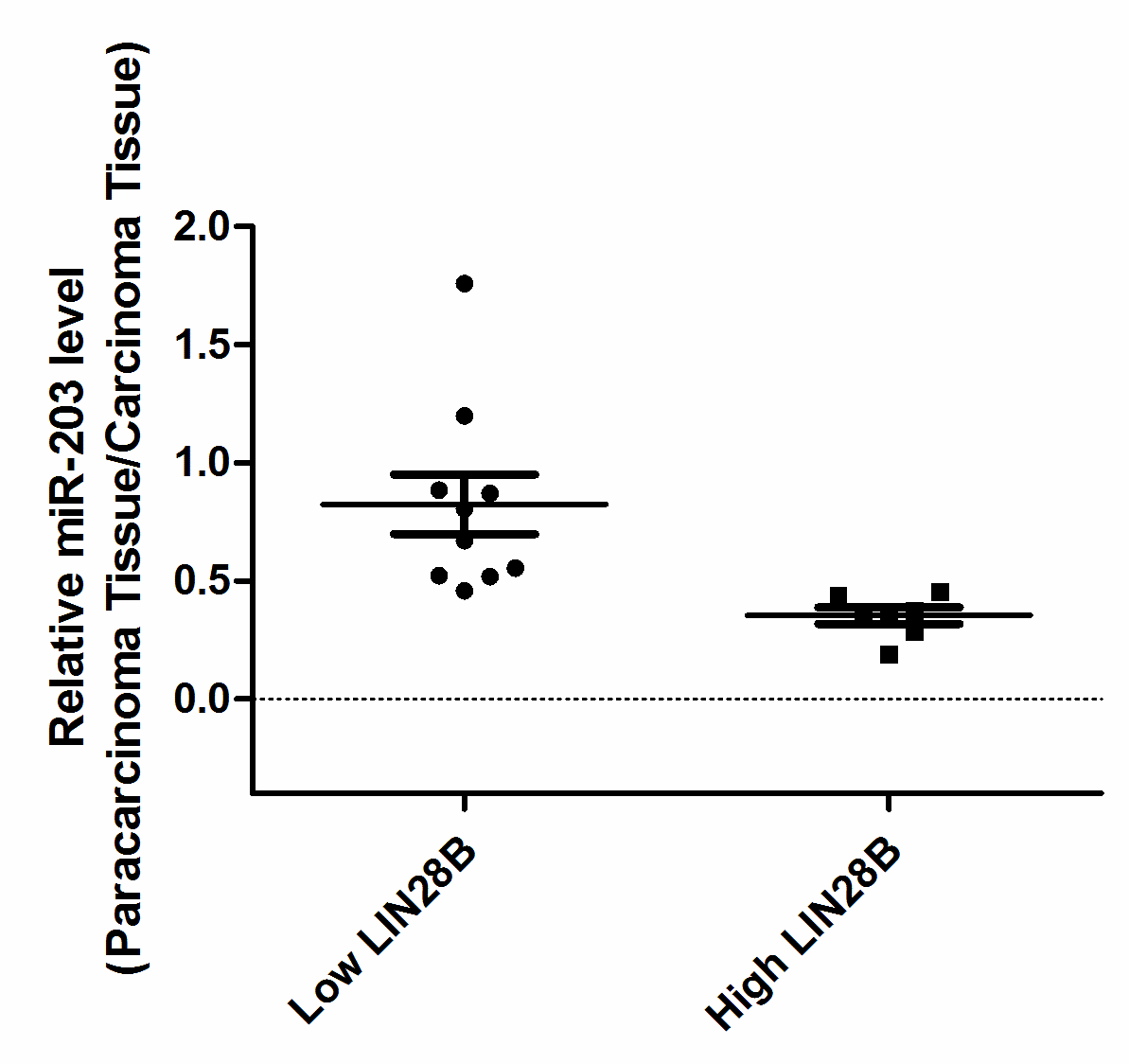
**

**Figure S3.** Quantitative RT-PCR analysis of the miR-203 levels in ten pairs of NSCLC samples with relatively Low LIN28B protein levels and seven pairs of NSCLC samples with considerably High LIN28B protein levels. The results are presented as the mean ± SE of three independent experiments (*P<0.05, **P<0.01, ***P<0.001).

**Figure S4.** Quantitative RT-PCR analysis of the let-7 levels in A549 cells treated with agomir control agomir plus control vector, control agomir plus LIN28B vector, miR-203 agomir plus control vector or miR-203 agomir plus LIN28B vector. The results are presented as the mean ± SE of three independent experiments (*P<0.05, **P<0.01, ***P<0.001).

**
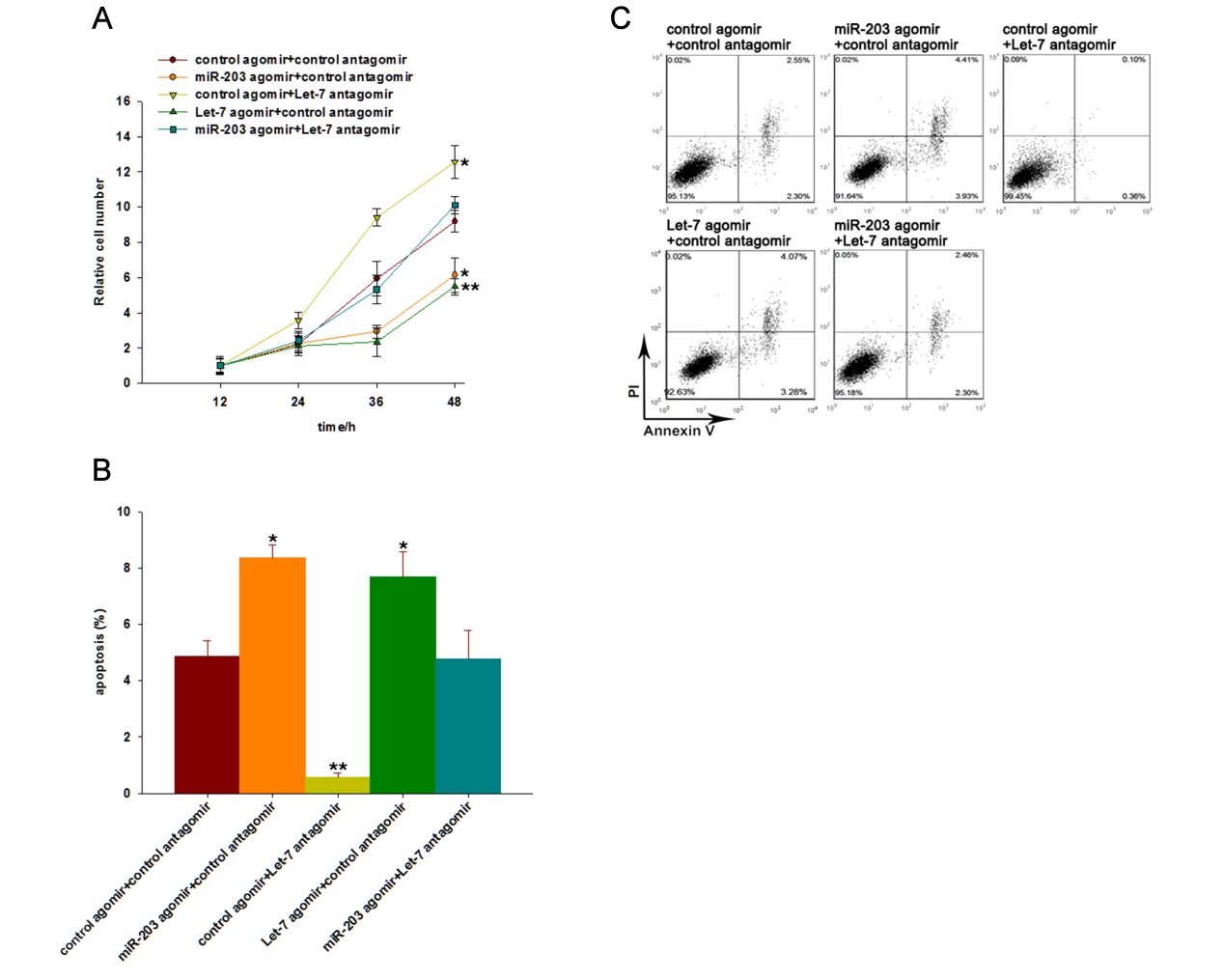
**

**Figure S5. The functions of miR203/LIN28B/let-7 axis.** (A) Relative cell proliferation in A549 cells after transfection with agomir control plus antagomir control, miR-203 agomir plus antagomir control, control agomir plus Let-7 antagomir, Let-7 agomir plus control antagomir, or miR-203 agomir plus Let-7 antagomir. (B) A549 cells were transfected with agomir control plus antagomir control, miR-203 agomir plus antagomir control, control agomir plus Let-7 antagomir, Let-7 agomir plus control antagomir, or miR-203 agomir plus Let-7 antagomir. The cell apoptosis profiles were analyzed using flow cytometry. The biparametric histogram shows cells in early (bottom right quadrant) and late apoptotic states (upper right quadrant). Viable cells are double negative (bottom left quadrant). B: quantitative analysis; C: representative image.The results are presented as the mean ± SE of three independent experiments (*P<0.05, **P<0.01, ***P<0.001).

**
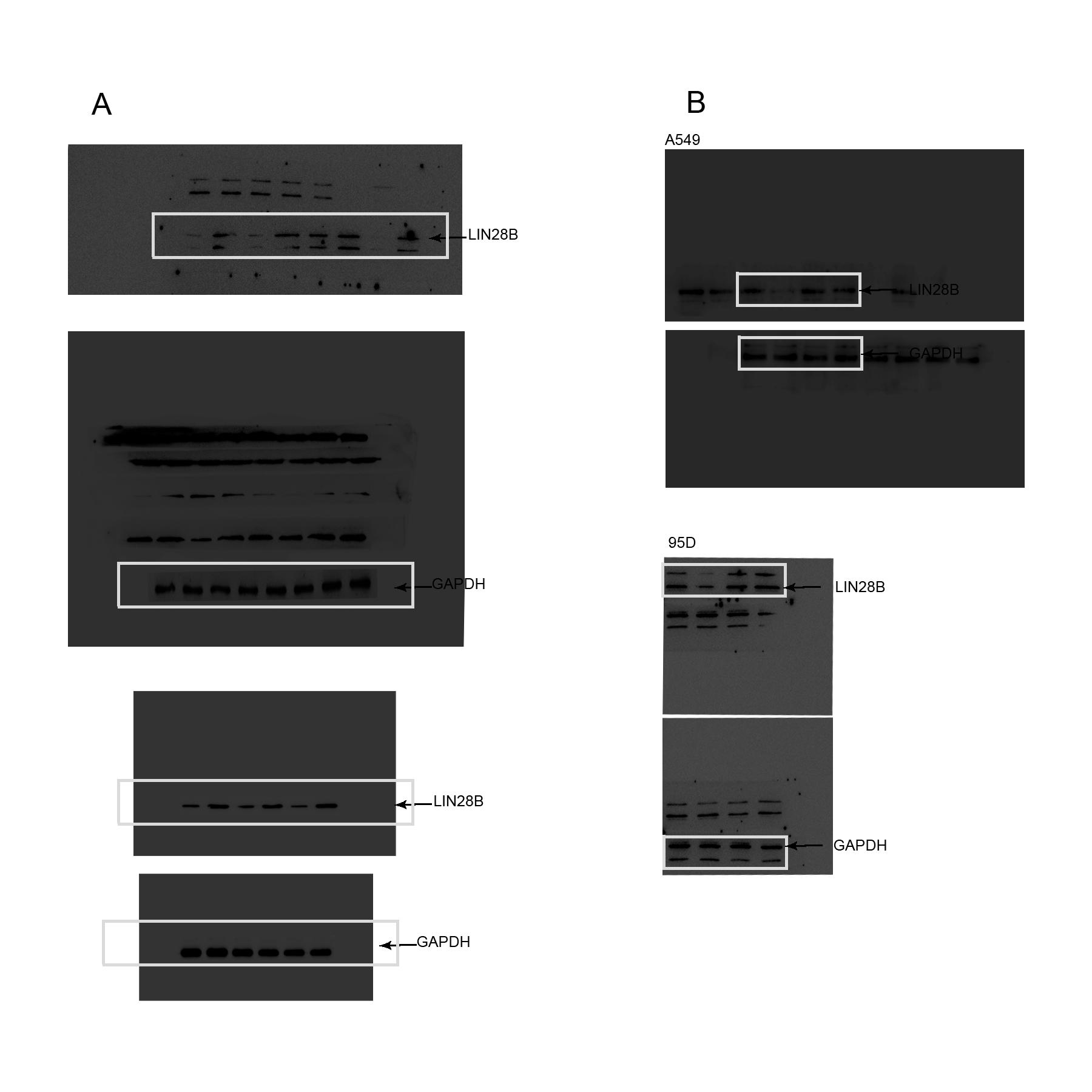
**

**Figure S6. Full gel images of Western blots and prestained size marker standards.** (A) The full-size images corresponding to those shown in Figure 1A and (B) Figure 3B. Chemiluminescence images stained with the indicated antibodies were sequentially captured with Tanon-5200s.

**Reference**

1. Chen T, Xu C, Chen J, Ding C, Xu Z, Li C and Zhao J. MicroRNA-203 inhibits cellular proliferation and invasion by targeting Bmi1 in non-small cell lung cancer. Oncol Lett. 2015; 9(6):2639-2646.

2. Zhong X, Li N, Liang S, Huang Q, Coukos G and Zhang L. Identification of microRNAs regulating reprogramming factor LIN28 in embryonic stem cells and cancer cells. J Biol Chem. 2010; 285(53):41961-41971.

3. Zhong K, Chen K, Han L and Li B. MicroRNA-30b/c inhibits non-small cell lung cancer cell proliferation by targeting Rab18. BMC Cancer. 2014; 14:703.

4. Zhong Z, Xia Y, Wang P, Liu B and Chen Y. Low expression of microRNA-30c promotes invasion by inducing epithelial mesenchymal transition in non-small cell lung cancer. Mol Med Rep. 2014; 10(5):2575-2579.

5. Guo Y, Chen Y, Ito H, Watanabe A, Ge X, Kodama T and Aburatani H. Identification and characterization of lin-28 homolog B (LIN28B) in human hepatocellular carcinoma. Gene. 2006; 384:51-61.

6. Johnson CD, Esquela-Kerscher A, Stefani G, Byrom M, Kelnar K, Ovcharenko D, Wilson M, Wang X, Shelton J, Shingara J, Chin L, Brown D and Slack FJ. The let-7 microRNA represses cell proliferation pathways in human cells. Cancer Res. 2007; 67(16):7713-7722.

7. Kumar MS, Erkeland SJ, Pester RE, Chen CY, Ebert MS, Sharp PA and Jacks T. Suppression of non-small cell lung tumor development by the let-7 microRNA family. Proc Natl Acad Sci U S A. 2008; 105(10):3903-3908.

8. Yu SL, Chen HY, Chang GC, Chen CY, Chen HW, Singh S, Cheng CL, Yu CJ, Lee YC, Chen HS, Su TJ, Chiang CC, Li HN, Hong QS, Su HY, Chen CC, et al. MicroRNA signature predicts survival and relapse in lung cancer. Cancer Cell. 2008; 13(1):48-57.

9. Chin LJ, Ratner E, Leng S, Zhai R, Nallur S, Babar I, Muller RU, Straka E, Su L, Burki EA, Crowell RE, Patel R, Kulkarni T, Homer R, Zelterman D, Kidd KK, et al. A SNP in a let-7 microRNA complementary site in the KRAS 3' untranslated region increases non-small cell lung cancer risk. Cancer Res. 2008; 68(20):8535-8540.

10. Lozoya T, Dominguez F, Romero-Ruiz A, Steffani L, Martinez S, Monterde M, Ferri B, Nunez MJ, AinhoaRomero E, Zamora O, Gurrea M, Sangiao-Alvarellos S, Vega O, Simon C, Pellicer A and Tena-Sempere M. The Lin28/Let-7 system in early human embryonic tissue and ectopic pregnancy. PLoS One. 2014; 9(1):e87698.

11. Sangiao-Alvarellos S, Manfredi-Lozano M, Ruiz-Pino F, Navarro VM, Sanchez-Garrido MA, Leon S, Dieguez C, Cordido F, Matagne V, Dissen GA, Ojeda SR, Pinilla L and Tena-Sempere M. Changes in hypothalamic expression of the Lin28/let-7 system and related microRNAs during postnatal maturation and after experimental manipulations of puberty. Endocrinology. 2013; 154(2):942-955.

12. Zhang B, Lu L, Zhang XJ, Ye WY, Wu J, Xi QL and Zhang XJ. Hsa-miR-132 Regulates Apoptosis in Non-Small Cell Lung Cancer Independent of Acetylcholinesterase. J Mol Neurosci. 2014; 53(3):335-344.

13. Li X, Zhang J, Gao L, McClellan S, Finan MA, Butler TW, Owen LB, Piazza GA and Xi Y. MiR-181 mediates cell differentiation by interrupting the Lin28 and let-7 feedback circuit. Cell Death Differ. 2012; 19(3):378-386.

14. Huang P, Ye B, Yang Y, Shi JX and Zhao H. MicroRNA-181 functions as a tumor suppressor in non-small cell lung cancer (NSCLC) by targeting Bcl-2. Tumor Biol. 2015; 36(5):3381-3387.

15. Borrego-Diaz E, Powers BC, Azizov V, Lovell S, Reyes R, Chapman B, Tawfik O, McGregor D, Diaz FJ, Wang X and Veldhuizen PV. A potential regulatory loop between Lin28B:miR212 in androgen-independent prostate cancer. Int J Oncol. 2014; 45(6):2421-2429.

16. Lu L, Zhang XJ, Zhang B, Wu J and Zhang XJ. Synaptic acetylcholinesterase targeted by microRNA-212 functions as a tumor suppressor in non-small cell lung cancer. Int J Biochem Cell B. 2013; 45(11):2530-2540.

17. Li Y, Zhang D, Chen C, Ruan Z and Huang Y. MicroRNA-212 displays tumor-promoting properties in non-small cell lung cancer cells and targets the hedgehog pathway receptor PTCH1. Mol Biol Cell. 2012; 23(8):1423-1434.

18. Incoronato M, Garofalo M, Urso L, Romano G, Quintavalle C, Zanca C, Iaboni M, Nuovo G, Croce CM and Condorelli G. miR-212 increases tumor necrosis factor-related apoptosis-inducing ligand sensitivity in non-small cell lung cancer by targeting the antiapoptotic protein PED. Cancer Res. 2010; 70(9):3638-3646.

19. Wang L, Zhang LF, Wu J, Xu SJ, Xu YY, Li DS, Lou JT and Liu MF. IL-1 beta-Mediated Repression of microRNA-101 Is Crucial for Inflammation-Promoted Lung Tumorigenesis. Cancer Research. 2014; 74(17):4720-4730.

20. Yan F, Shen N, Pang J, Xie D, Deng B, Molina JR, Yang P and Liu S. Restoration of miR-101 suppresses lung tumorigenesis through inhibition of DNMT3a-dependent DNA methylation. Cell Death Dis. 2014; 5.

21. Zhang JG, Guo JF, Liu DL, Liu QA and Wang JJ. MicroRNA-101 Exerts Tumor-Suppressive Functions in Non-small Cell Lung Cancer through Directly Targeting Enhancer of Zeste Homolog 2. J Thorac Oncol. 2011; 6(4):671-678.

22. Luo L, Zhang T, Liu H, Lv T, Yuan D, Yao Y, Lv Y and Song Y. MiR-101 and Mcl-1 in non-small-cell lung cancer: expression profile and clinical significance. Med Oncol. 2012; 29(3):1681-1686.

23. Faria AM, Sbiera S, Ribeiro TC, Soares IC, Mariani BM, Freire DS, de Sousa GR, Lerario AM, Ronchi CL, Deutschbein T, Wakamatsu A, Alves VA, Zerbini MC, Mendonca BB, Fragoso MC, Latronico AC, et al. Expression of LIN28 and its regulatory microRNAs in adult adrenocortical cancer. Clin Endocrinol (Oxf). 2015; 82(4):481-488.

24. La Torre A, Georgi S and Reh TA. Conserved microRNA pathway regulates developmental timing of retinal neurogenesis. Proc Natl Acad Sci U S A. 2013; 110(26):E2362-2370.

25. Garofalo M, Quintavalle C, Di Leva G, Zanca C, Romano G, Taccioli C, Liu CG, Croce CM and Condorelli G. MicroRNA signatures of TRAIL resistance in human non-small cell lung cancer. Oncogene. 2008; 27(27):3845-3855.

26. Du L, Schageman JJ, Subauste MC, Saber B, Hammond SM, Prudkin L, Wistuba, II, Ji L, Roth JA, Minna JD and Pertsemlidis A. miR-93, miR-98, and miR-197 regulate expression of tumor suppressor gene FUS1. Mol Cancer Res. 2009; 7(8):1234-1243.
